# Motor Vehicle Protective Device Usage Associated with Decreased Rate of Flail Chest: A Retrospective Database Analysis

**DOI:** 10.3390/medicina59112046

**Published:** 2023-11-20

**Authors:** Aria Bassiri, Avanti Badrinathan, Sami Kishawi, Jillian Sinopoli, Philip A. Linden, Vanessa P. Ho, Christopher W. Towe

**Affiliations:** 1 Department of Surgery, Division of Thoracic and Esophageal Surgery, University Hospitals Cleveland Medical Center, Cleveland, OH 44106, USA; aria.bassiri@uhhospitals.org (A.B.); avanti.badrinathan@uhhospitals.org (A.B.); sami.kishawi@uhhospitals.org (S.K.); jillian.sinopoli@uhhospitals.org (J.S.); philip.linden@uhhospitals.org (P.A.L.); 2 Department of Surgery, Division of Trauma, Critical Care, Burns, and Acute Care Surgery, MetroHealth Medical Center, Cleveland, OH 44109, USA; vho@metrohealth.org

**Keywords:** flail chest, rib fracture, seatbelt, airbag, vehicle protective equipment, motor vehicle collision

## Abstract

*Background and Objectives*: Protective equipment, including seatbelts and airbags, have dramatically reduced the morbidity and mortality rates associated with motor vehicle collisions (MVCs). While generally associated with a reduced rate of injury, the effect of motor vehicle protective equipment on patterns of chest wall trauma is unknown. We hypothesized that protective equipment would affect the rate of flail chest after an MVC. *Materials and Methods*: This study was a retrospective analysis of the 2019 iteration of the American College of Surgeons Trauma Quality Program (ACS-TQIP) database. Rib fracture types were categorized as non-flail chest rib fractures and flail chest using ICD-10 diagnosis coding. The primary outcome was the occurrence of flail chests after motor vehicle collisions. The protective equipment evaluated were seatbelts and airbags. We performed bivariate and multivariate logistic regression to determine the association of flail chest with the utilization of vehicle protective equipment. *Results*: We identified 25,101 patients with rib fractures after motor vehicle collisions. In bivariate analysis, the severity of the rib fractures was associated with seatbelt type, airbag status, smoking history, and history of cerebrovascular accident (CVA). In multivariate analysis, seatbelt use and airbag deployment (OR 0.76 CI 0.65–0.89) were independently associated with a decreased rate of flail chest. In an interaction analysis, flail chest was only reduced when a lap belt was used in combination with the deployed airbag (OR 0.59 CI 0.43–0.80) when a shoulder belt was used without airbag deployment (0.69 CI 0.49–0.97), or when a shoulder belt was used with airbag deployment (0.57 CI 0.46–0.70). *Conclusions*: Although motor vehicle protective equipment is associated with a decreased rate of flail chest after a motor vehicle collision, the benefit is only observed when lap belts and airbags are used simultaneously or when a shoulder belt is used. These data highlight the importance of occupant seatbelt compliance and suggest the effect of motor vehicle restraint systems in reducing severe chest wall injuries.

## 1. Introduction

Motor vehicle collisions (MVCs) remain a prevalent and critical public health concern, contributing significantly to the global burden of trauma-related injuries and fatalities. MVCs account for nearly 1.35 million deaths every year worldwide, making it the eighth leading cause of death in all age groups globally [1]. Studies of injury patterns demonstrate that MVCs are more likely to lead to severe chest injuries compared to other mechanisms [2]. Thoracic trauma accounts for nearly 25% of trauma-related mortality, with approximately 40% presenting with rib fractures after blunt chest trauma [3,4,5]. Of this subset, approximately 6% of patients present with flail chest [4,5]. Among the myriad of injuries resulting from MVCs, rib fractures and flail chests represent common and often severe consequences, contributing substantially to morbidity and mortality rates. While most isolated rib fractures are often treated non-operatively, flail chest is often associated with higher morbidity and mortality rates [5,6]. In turn, patients with increasing severity of rib fracture patterns, such as flail chest, are at a higher risk of pulmonary complications, prolonged ventilator use, and longer ICU and hospital stays [5,6,7]. As previous studies have drawn attention to the significance of the patient population affected by these injuries, there is a pressing need to better understand and mitigate the associated risks.

Seatbelt utilization and the incorporation of airbags in contemporary vehicles mark significant milestones in occupant safety. Protective vehicle equipment, such as seatbelts and airbags, have dramatically reduced the morbidity and mortality rates associated with motor vehicle collisions. The National Highway Traffic Safety Administration estimates that in 2017, 14,955 lives of occupants aged 5 and older were saved by seatbelts, and 2790 lives of those aged 13 and older were saved by frontal airbags [8]. Seatbelt use is associated with less severe injuries and lower in-hospital mortality [9,10,11]. Although generally associated with reduced rates of injury, mortality, and morbidity, protective equipment has been shown to alter the injury patterns in abdominal trauma and orthopedic injuries [12]. Restrained occupants demonstrated a significant reduction in the severity of injury in all body areas, lower mortality rates, shorter hospital stays, and a lower number of operations. However, these occupants also had significantly higher rates of hollow viscos injury compared to unrestrained occupants [12,13]. The use of airbags alone is associated with a decrease in injuries to the brain, face, cervical spine, thorax, and abdomen [14]. The greatest reduction in injuries has been noted when seatbelts are used in conjunction with airbags, with the exception of an increase in extremity fractures with airbag deployment [14]. Understanding the intricate relationship between seatbelt use, airbag deployment, and traumatic injuries is paramount for refining injury prevention strategies and enhancing vehicle safety systems.

To our knowledge, there have been no studies evaluating the impact of vehicle protective equipment, such as seatbelts and airbags, on the pattern and severity of rib fractures. As such, the effect of motor vehicle protective equipment on the patterns and severity of rib fractures is unknown. The aim of this study is to evaluate the impact of vehicle protective equipment on chest wall injury patterns, specifically flail chest. We hypothesized that the utilization of vehicle protective equipment would decrease the rate of flail chest among patients presenting with rib fractures after MVCs.

## 2. Materials and Methods

**Figure 1:**
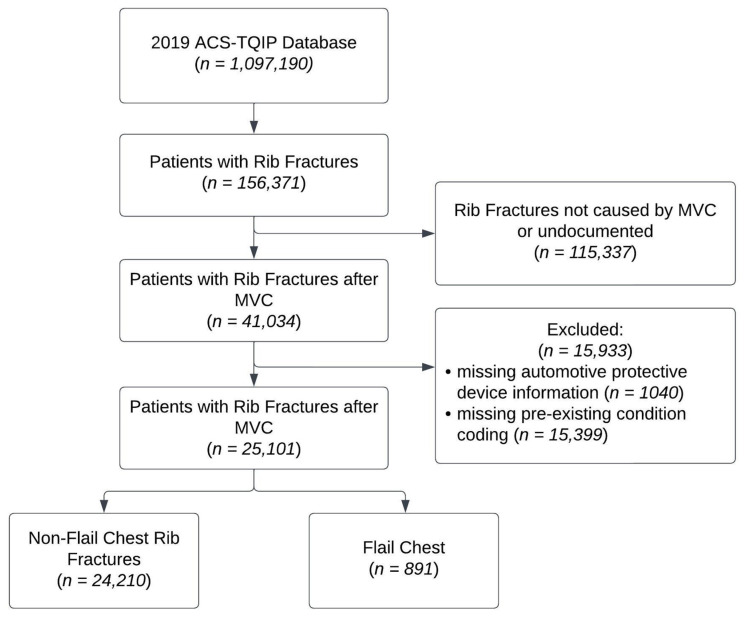
Retrospective database analysis of the 2019 American College of Surgeons Trauma Quality Program (ACS-TQIP) database of patients presenting with rib fractures after motor vehicle collision (MVC).

We performed a retrospective database analysis of the 2019 American College of Surgeons Trauma Quality Program (ACS-TQIP) database [15]. This is a large, deidentified database of trauma registry information from participating trauma centers. We identified all adult patients ≥ 16 years of age, without missing data, who sustained rib fractures secondary to a blunt injury mechanism, as defined using ICD-10 external cause coding (Figure 1). Study design was created using a Strengthening the Reporting of Observational Studies in Epidemiology (STROBE) checklist (Supplementary Materials—Table S1) [16]. Further grouping was performed based on external cause coding to identify patients who were involved in MVCs. Patients were further categorized by rib fracture type (single rib, multiple ribs, and flail chest) using ICD-10 diagnosis coding. A flail chest is defined as three or more consecutive rib fractures in two or more locations, creating a flail segment. Pre-existing conditions were grouped into diagnoses using Clinical Classifications Software Refined v2023.1 for ICD-10 [17]. Abbreviated Injury Scale (AIS) scoring was calculated using the ICD-PIC R package to determine maximum AIS scores per body region [18,19].

Safety device information, such as seatbelt usage, was defined in the TQIP database as the device used by the patient at time of injury. Lap belt was used to define lap belt usage as well as unspecified seatbelt restraints found on patients at the time of injury via TQIP [20]. Shoulder belt group was defined as having the presence of both a lap and shoulder belt at the time of injury. Airbag deployment was defined according to the location of airbag (front, side, or other) or non-deployment. Our primary goal was to determine the occurrence of flail chest injury with other variables of interest, including age, gender, pre-existing conditions, seatbelt type used, airbag type deployed, and AIS scoring by body region.

Descriptive analyses were performed using Chi-square testing for categorical variables and Wilcoxon rank-sum testing for continuous variables. All continuous variables were assumed to be non-normally distributed, and all analyses of continuous variables were conducted accordingly. Multivariate logistic regression was performed using clinically salient variables to determine the odds of flail chest injury. A further multivariate regression analysis of the interaction between seatbelt and airbag types was performed to determine the synergistic effects of protective devices on the severity of rib fractures. Results are presented as odds ratio (OR) with 95% confidence intervals (95% CI). Findings were considered significant for *p* ≤ 0.05. All analyses were performed using STATA SE/17 (StataCorp, College Station, TX, USA). This study was determined to be exempt from receiving an Institutional Review Board review and did not require informed consent for the use of deidentified data.

## 3. Results

### 3.1. Patient Characteristics

The analysis includes 25,101 patients with rib fractures after motor vehicle collisions who met the inclusion criteria. This includes 4704 single rib fractures (18.7%), 19,506 multiple rib fractures (77.7%), and 891 flail chest injuries (3.6%). The demographic characteristics of the patients are shown in Table 1. In bivariate analysis, the type of seatbelt used and the airbag status were associated with flail chest. Patients with flail chest were more likely to be older (*p* = 0.0368) and to have higher maximum AIS scores in the head/neck, face, extremities, and abdominal regions (*p* < 0.001). Furthermore, patients with flail chest had higher rates of a history of smoking (5.6% vs. 4.2%, *p* = 0.040) and a history of cerebrovascular accidents (CVA) (1.46% vs. 0.61%, *p* = 0.002) compared to patients with non-flail rib fractures.

### 3.2. Seatbelt Use and Airbag Deployment

A multivariate logistic regression was performed to investigate the risk of flail chest after an MVC (Table 2). Increasing age was independently associated with flail chest (OR 1.01, 1.01–1.02, *p* < 0.001). Compared to patients with no seatbelt, patients with a lap belt (OR 0.72, CI 0.57–0.90) or shoulder belt (OR 0.73, CI 0.63–0.86) were at lower risks of flail chest. Similarly, airbag deployment was associated with a lower risk of flail chest (OR 0.76, CI 0.65–0.89). In contrast, an increased risk of flail chest was associated with patients with non-chest maximum AIS scores in their head/neck (OR 1.13, CI 1.07–1.18), extremity (OR 1.22, CI 1.15–1.30), and abdomen (OR 1.38, CI 1.31–1.45) regions. It is worth noting that a history of CVA was associated with an increased risk of flail chest (OR 2.17, CI 1.21–3.88).

### 3.3. Synergistic Effects of Seatbelts and Airbags

**Figure 2:**
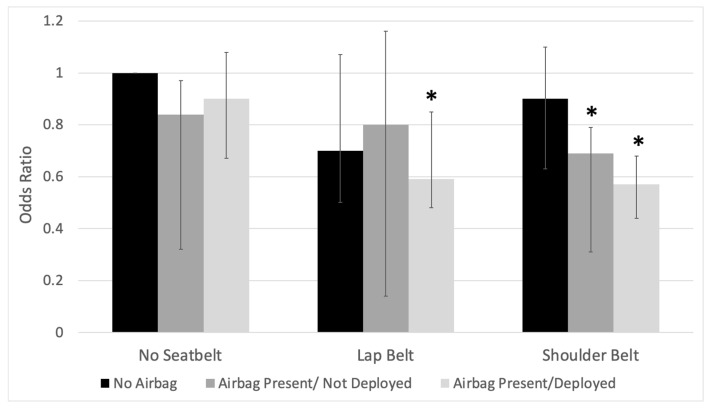
Association of risk of flail chest and vehicle protective equipment amongst patients with rib fractures after motor vehicle collisions in the 2019 ACS = TQIP database. Odds ratio from multivariate model was adjusted for age, sex, vehicle protective equipment, maximum abbreviated injury scale, history of smoking, and history of cerebrovascular accident. Reference group—no seatbelt/no airbags. Asterisk (*) denotes significance *p* ≤ 0.05.

A further multivariable logistic regression analysis of the interaction between seatbelt and airbag type was performed to determine the synergistic effects between protective devices (Table 3). In this analysis, increasing age was associated with an increased risk of flail chest (OR 1.01, CI 1.01–1.02). Interestingly, the risk of flail chest was only reduced when a lap belt was used in combination with the deployed airbag (OR 0.59, CI 0.43–0.80) when a shoulder belt was used when airbags were present but not deployed (OR 0.69, CI 0.49–0.97), or when a shoulder belt was used with an airbag deployed (OR 0.57, CI 0.46–0.70) (Figure 2). Like our previous multivariate analysis, a history of CVA (OR 2.16, CI 1.21–3.87) and maximum AIS in the head/neck (OR 1.13, CI 1.07–1.18), extremities (OR 1.22, CI 1.15–1.30), and abdomen (OR 1.38, CI 1.31–1.45) were associated with an increased risk of flail chest.

## 4. Discussion

This is a retrospective national database analysis demonstrating the impact of vehicle protective equipment on patterns of chest wall injuries in patients presenting with rib fractures after MVCs. More specifically, this study further elucidates the complex relationship between the use of protective devices, such as seatbelts and airbags, and the risk of flail chest after MVCs. Although motor vehicle protective equipment utilization is associated with a decreased rate of flail chest after motor vehicle collision, there is only a benefit when lap belts and airbags are used simultaneously, or a shoulder belt is used (with or without airbag deployment). There was no difference in the severity of rib fractures among unbelted passengers when airbags were deployed. Furthermore, an increase in age, a history of smoking, and a history of CVA were associated with an increased risk of flail chest. Of note, patients with maximum AIS scores in the head/neck, extremities, or abdomen were associated with a higher likelihood of flail chest among patients presenting with rib fractures after MVCs. The findings of this study reveal essential insights into the reduction in flail chest rates when specific protective devices are employed and underscore the importance of identifying high-risk individuals who may benefit from improved preventative measures and management.

Flail chest can lead to chest wall instability, asynchronous movement of the flail segment, and paradoxical chest wall motion [4,5,6,21]. As such, this leads to deformity of the chest wall and a loss of thoracic volume. This will ultimately cause atelectasis, decreased lung volume, dyspnea, and chronic pain [5,6,21]. Given the severity of these injuries, flail chest is associated with higher morbidity (intensive care unit (ICU) admission, mechanical ventilation, need for chest tubes, tracheostomy, acute respiratory distress syndrome (ARDS), and sepsis) as well as higher mortality [6]. Current treatment guidelines for flail chest injuries include a multimodal pain regimen, chest physiotherapy, pulmonary toilet, positive pressure ventilation, and, in certain circumstances, rib stabilization [6,22,23]. Given the significant morbidity and mortality risks associated with flail chest in patients after blunt thoracic trauma, a focus on preventative measures is imperative.

Vehicle protective equipment, including seatbelts and airbags, has been associated with differences in injury patterns compared to patients who do not use protective equipment [11]. In a prospective study performed on injury patterns and the impact of seat belt use, unrestrained patients were more likely to have a higher AIS score in the thorax, back, and lower extremities compared to restrained passengers and were more likely to have lower GCS scores or undergo surgical operations [13]. A similar study evaluating mechanisms of injury and restraint use demonstrated decreases in brain injuries in restrained passengers involved in frontal MVCs while having found no difference in lateral MVCs [24]. Furthermore, they suggested seatbelt use did not protect against lung, liver, spleen, pelvis, and lower extremity injury, suggesting that the direction of a crash appeared to play a more significant role. Lower extremity injuries were higher in frontal crashes, while pelvic injuries were associated with lateral crashes [24]. Here, we demonstrated that among patients presenting with rib fractures after MVCs, seatbelt use and airbag deployment were independently associated with decreased rates of flail chest. In our interaction multivariate analysis, we demonstrated the synergistic relationship between seatbelt use and airbag deployment. However, it should be noted that this relationship differed based on the seatbelt type used. Patients with lap belts had lower rates of flail chests only when airbags were deployed. Furthermore, when a shoulder belt was utilized alone with an airbag present (deployed or not deployed), the risk of flail chest decreased. This further demonstrates the significance of the type of restraint used and the synergistic benefit of vehicle protective equipment on impacting chest wall injury pattern and severity. We propose that the biomechanical differences in forces experienced by the patient, with and without protective devices, play a significant role in the injury the patients may sustain. Thus, this synergistic effect underscores the necessity of public education on the proper and comprehensive use of safety features in vehicles.

As previously highlighted, flail chest is associated with significant morbidity and mortality. As such, identifying patients at risk may offer valuable insights into guiding the management of these patients for more favorable outcomes. In the study of patients with rib fractures after MVCs, it is important to consider underlying risk factors associated with these injuries. Diminished bone density is associated with an increased risk of bone fractures [25]. Particularly after blunt thoracic trauma, previous literature has demonstrated that patients with lower bone mineral density have higher rates of rib fractures compared to patients with normal bone mineral density [26]. There may be many factors associated with diminished bone density. An increase in age is strongly associated with diminished bone density and thus more susceptible to fractures [25,26,27]. Furthermore, other factors, such as a history of smoking, have been linked to reduced bone density and a higher risk of bone fracture [28]. In our cohort, an increase in age and a history of smoking were independently associated with an increased risk of flail chest amongst patients presenting with rib fractures after MVCs. While we were unable to analyze bone density among our cohort, as it is not included in the ACS-TQIP database, we speculate that both an increase in age and a history of smoking are associated with decreased bone density. In turn, these patients are more likely to sustain more severe rib fracture patterns, such as flail chest, after MVCs. Interestingly, we found that patients with a history of CVA are also independently associated with an increased risk of flail chest. Patients with a history of CVA may exhibit lower bone density due to underuse or secondary motor deficits [29]. In contrast, there is also an increased risk of stroke among patients with diminished bone density [30]. In our cohort, we are unable to assess bone density as it relates to a history of CVA. However, a history of CVA may provide insights into a patient’s bone density. More specifically, we hypothesize that a history of CVA may be indicative of diminished bone density in these patients, subsequently leading to an increased risk of flail chest after MVCs. Lastly, our analysis demonstrated that patients with flail chest had maximum AIS scores in the head/neck, extremities, and abdomen. Collectively, this information can be used by providers for more expeditious identification of these patients at risk to help guide management plans for improved outcomes. Future studies should focus on identifying other underlying factors that may be associated with an increased risk of more severe fracture patterns, such as flail chest, after MVCs.

The findings of our study have significant implications for clinical practice, particularly in a trauma care setting. Healthcare professionals should consider the observed synergistic effects of seatbelts and airbags in reducing incidences of flail chest injuries among patients with rib fractures after MVCs. Trauma care teams may need to emphasize the importance of proper seatbelt usage and advocate for comprehensive occupant protection. Furthermore, this information can guide emergency medical personnel in providing more targeted and effective interventions for patients at risk of severe chest injuries. Additionally, these findings further highlight the importance of public health initiatives and education campaigns focused on vehicular safety. Raising awareness about the specific protective devices, such as seatbelts and airbags, that contribute to a decreased severity of chest wall trauma can have a profound impact on reducing the overall morbidity and mortality associated with MVCs. Public health organizations should consider incorporating these findings into educational materials and campaigns aimed at encouraging proper seatbelt use and advocating for the widespread adoption of safety features in vehicles. Recognizing the multidisciplinary nature of addressing chest trauma after MVCs, collaboration across the medical, engineering, and public health disciplines is indispensable. A holistic approach, encompassing healthcare professionals, engineers specializing in vehicle safety, and public health experts, can combine efforts to formulate comprehensive strategies. Integrating engineering insights into vehicle design with medical expertise in trauma care ensures a more cohesive and effective approach to injury prevention and patient management.

### Limitations

While the benefit of seatbelts in the alteration of injury patterns has been heavily advocated, to our knowledge, no study has evaluated the impact of other vehicle protective equipment, like airbags, in conjunction with seatbelt use. Moreover, no study has evaluated the impact of these devices on patterns of chest wall trauma. We recognize that this study has several limitations. The ACS-TQIP database is a deidentified administrative database and, as such, individual entries cannot be verified for accuracy. As a result, errors in billing and coding information cannot be addressed. Patients were identified using ICD-10 coding, and the study population could potentially be impacted by errors in coding. Furthermore, we were unable to evaluate other factors that may increase the risk of more severe rib fractures, such as bone density. While the ACS-TQIP database provides a select history of underlying comorbidities, more granular information regarding pertinent patients’ medical history would allow for a more comprehensive analysis of risk factors associated with the severity of injuries after MVCs. Protective device information was provided by the TQIP database and defined as protective device usage noted at the time of injury by emergency personnel. As a result, potential damage or patient factors could misidentify the device use. In addition, unspecified restraints were all categorized as lap belts, though they may not have been lap belts. Furthermore, information regarding crash severity, the direction of the collision, the speed of the collision, and vehicle information were not available for analysis in the ACS-TQIP database. Such factors have previously been demonstrated to be associated with altered injury severity and patterns in patients sustaining injuries through MVCs. Future studies should focus on developing and utilizing a database that contains both comprehensive clinical information on patients as well as more granular details pertaining to the MVCs. Despite these limitations, we believe that our study captures the impact of multiple vehicle protective devices on the severity of chest wall trauma patterns in patients presenting with rib fractures after MVCs.

## 5. Conclusions

In conclusion, the use of vehicle protective equipment reduces the rate of flail chest amongst patients presenting with rib fractures after MVCs. The benefits have only been observed with lap belts and simultaneous airbag deployment or shoulder belts (with or without airbag deployment). These data highlight the importance of occupant seatbelt compliance and suggest the connection between the use of motor vehicle protective equipment and the reduction in severe chest wall injuries. By identifying patient risk factors, clinicians can better recognize those at higher risk of flail chest following MVCs, ultimately leading to improved patient care and outcomes. Future studies should aim to explore additional factors that may contribute to more severe rib fracture patterns and expand our understanding of the biomechanical aspects of these injuries during MVCs.

## Figures and Tables

**Table 1:** Relationship of clinical and demographic factors to the occurrence of flail chest among patients with rib fractures after motor vehicle collision as identified in the 2019 ACS-TQIP database. Data shown as *n* (%) for categorical variables or Median (IQR) for continuous variables. AIS—Abbreviated Injury Scale. CVA—cerebrovascular accident. IQR—Interquartile range.

Variable	Non-Flail Chest Rib Fractures (*n* = 24,210)	Flail Chest (*n* = 891)	*p* Value
Age (Median)	58 (40–71)	59 (44–71)	0.0368
Sex			0.481
Female	10,994 (45.4%)	394 (44.22%)	
Male	12,213 (54.6%)	497 (55.8%)	
Race			
White	18,653 (77.0%)	703 (78.9%)	0.196
Black	3174 (13.1%)	111 (12.5%)	0.571
Asian	423 (1.7%)	15 (1.7%)	0.887
Ethnicity			0.221
Hispanic or Latino	2167 (9.3%)	69 (8.1%)	
Not Hispanic or Latino	21,121 (90.7%)	786 (91.9%)	
Seatbelt Type			<0.001
No seatbelt	7304 (30.2%)	350 (39.3%)	
Lap belt only	3366 (13.9%)	106 (11.9%)	
Shoulder belt	13,540 (55.9%)	435 (48.8%)	
Airbag Status			<0.001
No airbag present	6254 (25.8%)	296 (33.2%)	
Airbag present not deployed	2683 (11.1%)	94 (10.6%)	
Airbag deployed	15,273 (63.1%)	501 (56.2%)	
Body region maximum AIS (median IQR)			
Head/Neck	1 (0–1)	1 (0–3)	<0.001
General	0 (0–0)	0 (0–0)	0.0753
Face	0 (0–0)	0 (0–1)	0.003
Extremities	1 (0–1)	1 (1–2)	<0.001
Abdomen	1 (0–1)	1 (0–2)	<0.001
History of Smoking	1017 (4.2%)	50 (5.61%)	0.040
History of CVA	147 (0.61%)	13 (1.46%)	0.002

**Table 2:** Multivariate logistic regression of factors associated with flail chest injuries among patients with rib fractures after motor vehicle collisions in the 2019 ACS-TQIP database. AIS—Abbreviated Injury Scale. CVA—cerebrovascular accident. IQR—Interquartile range.

Variable	Odds Ratio	95% Confidence Interval	*p* Value
Age	1.01	1.01–1.02	<0.001
Male sex	1.03	0.89–1.18	0.701
Seatbelt Type			
No seatbelt	reference		
Lap belt only	0.72	0.57–0.90	0.004
Shoulder belt	0.73	0.63–0.86	<0.001
Airbag			
No airbag present	reference		
Airbag present not deployed	0.86	0.67–1.09	0.208
Airbag deployed	0.76	0.65–0.89	0.001
Body region maximum AIS			
Head/Neck	1.13	1.07–1.18	<0.001
General	1.04	0.93–1.16	0.500
Face	1.07	0.95–1.20	0.255
Extremities	1.22	1.15–1.30	<0.001
Abdomen	1.38	1.31–1.45	<0.001
History of Smoking	1.29	0.96–1.73	0.096
History of CVA	2.17	1.21–3.88	0.009

**Table 3:** Multivariate interaction analysis of factors associated with flail chest injuries among patients with rib fractures after motor vehicle collisions in the 2019 ACS-TQIP database. AIS—Abbreviated Injury Scale. CVA—cerebrovascular accident. IQR—Interquartile range.

Variable	Odds Ratio	95% Confidence Interval	*p* Value
Age	1.01	1.01–1.02	<0.001
Male sex	1.03	0.89–1.18	0.720
Seatbelt Type and airbag status (Interaction variable)			
No seatbelt/no airbag	reference		
No seatbelt/airbag present, not deployed	0.84	0.57–1.22	0.358
No seatbelt/airbag deployed	0.90	0.72–1.13	0.377
Lap belt/no airbag	0.70	0.47–1.04	0.079
Lap belt/airbag present, not deployed	0.80	0.44–1.46	0.468
Lap belt/airbag deployed	0.59	0.43–0.80	0.001
Shoulder belt/no airbag	0.90	0.70–1.17	0.448
Shoulder belt/airbag present, not deployed	0.69	0.49–0.97	0.030
Shoulder belt/airbag deployed	0.57	0.46–0.70	<0.001
Body region maximum AIS			
Head/Neck	1.13	1.07–1.18	<0.001
General	1.04	0.93–1.16	0.519
Face	1.07	0.95–1.20	0.257
Extremities	1.22	1.15–1.30	<0.001
Abdomen	1.38	1.30–1.45	<0.001
History of Smoking	1.28	0.95–1.73	0.103
History of CVA	2.16	1.21–3.87	0.009

## Data Availability

Upon request, the corresponding author can provide the data utilized in this study.

## Supplementary Materials

The following supporting information can be downloaded at: https://www.mdpi.com/article/10.3390/medicina59112046/s1. Table S1: Strengthening the Reporting of Observational Studies in Epidemiology (STROBE) Statement checklist of items that should be included in reports of observational studies for study titled “Motor Vehicle Protective Device Usage Associated with Decreased Rate of Flail Chest: A Retrospective Database Analysis”.
