# Supplementary material for: Motor Vehicle Protective Device Usage Associated with Decreased Rate of Flail Chest: A Retrospective Database Analysis
Source: Medicina (Kaunas). 2023 Nov 20;59(11):2046. doi: 10.3390/medicina59112046 (PMC10673139; doi:10.3390/medicina59112046)
Supplement: Supplementary file 1 [file medicina-59-02046-s001.zip › medicina-2655793-supplementary.pdf]

Table S1. Strengthening the Reporting of Observational Studies in Epidemiology (STROBE) Statement checklist of items that should be included in reports of observational studies for study titled “Motor Vehicle Protective Device Usage Associated with Decreased Rate of Flail Chest: A Retrospective Database Analysis”

	Item No	Recommendation	Page No
**Title and abstract**	1	(*a*) Indicate the study’s design with a commonly used term in the title or the abstract	Abstract
		(*b*) Provide in the abstract an informative and balanced summary of what was done and what was found	Abstract
**Introduction**			
Background/rationale	2	Explain the scientific background and rationale for the investigation being reported	Manuscript, Introduction
Objectives	3	State specific objectives, including any prespecified hypotheses	Manuscript, Introduction
**Methods**			
Study design	4	Present key elements of study design early in the paper	Manuscript, Methods, Figure 1
Setting	5	Describe the setting, locations, and relevant dates, including periods of recruitment, exposure, follow-up, and data collection	Manuscript, Methods
Participants	6	(*a*) *Cohort study*—Give the eligibility criteria, and the sources and methods of selection of participants. Describe methods of follow-up<br>*Case-control study*—Give the eligibility criteria, and the sources and methods of case ascertainment and control selection. Give the rationale for the choice of cases and controls<br>*Cross-sectional study*—Give the eligibility criteria, and the sources and methods of selection of participants	Manuscript, Methods
		(*b*) *Cohort study*—For matched studies, give matching criteria and number of exposed and unexposed<br>*Case-control study*—For matched studies, give matching criteria and the number of controls per case	
Variables	7	Clearly define all outcomes, exposures, predictors, potential confounders, and effect modifiers. Give diagnostic criteria, if applicable	Manuscript, Methods
Data sources/ measurement	8*	For each variable of interest, give sources of data and details of methods of assessment (measurement). Describe comparability of assessment methods if there is more than one group	Manuscript, Methods
Bias	9	Describe any efforts to address potential sources of bias	Manuscript, Methods
Study size	10	Explain how the study size was arrived at	Manuscript, Methods, Figure 1

Quantitative variables	11	Explain how quantitative variables were handled in the analyses. If applicable, describe which groupings were chosen and why	Manuscript, Methods
Statistical methods	12	(*a*) Describe all statistical methods, including those used to control for confounding	Manuscript, Methods
		(*b*) Describe any methods used to examine subgroups and interactions	Manuscript, Methods
		(*c*) Explain how missing data were addressed	Manuscript, Methods
		(*d*) *Cohort study*—If applicable, explain how loss to follow-up was addressed<br>*Case-control study*—If applicable, explain how matching of cases and controls was addressed<br>*Cross-sectional study*—If applicable, describe analytical methods taking account of sampling strategy	Manuscript, Methods
		(*e*) Describe any sensitivity analyses	

Continued on next page

**Results**			
Participants	13*	(a) Report numbers of individuals at each stage of study—eg numbers potentially eligible, examined for eligibility, confirmed eligible, included in the study, completing follow-up, and analysed	
		(b) Give reasons for non-participation at each stage	
		(c) Consider use of a flow diagram	Supplemental Content Figure 1
Descriptive data	14*	(a) Give characteristics of study participants (eg demographic, clinical, social) and information on exposures and potential confounders	Manuscript, Results; Table 1
		(b) Indicate number of participants with missing data for each variable of interest	
		(c) *Cohort study*—Summarise follow-up time (eg, average and total amount)	
Outcome data	15*	*Cohort study*—Report numbers of outcome events or summary measures over time	
		Case-control study—Report numbers in each exposure category, or summary measures of exposure	
		Cross-sectional study—Report numbers of outcome events or summary measures	Manuscript, Results
Main results	16	(*a*) Give unadjusted estimates and, if applicable, confounder-adjusted estimates and their precision (eg, 95% confidence interval). Make clear which confounders were adjusted for and why they were included	Manuscript, Results, Unmatched Analysis
		(*b*) Report category boundaries when continuous variables were categorized	
		(*c*) If relevant, consider translating estimates of relative risk into absolute risk for a meaningful time period	
Other analyses	17	Report other analyses done—eg analyses of subgroups and interactions, and sensitivity analyses	Manuscript, Results, Multivariable interaction analysis
**Discussion**			
Key results	18	Summarise key results with reference to study objectives	Manuscript, Discussion
Limitations	19	Discuss limitations of the study, taking into account sources of potential bias or imprecision. Discuss both direction and magnitude of any potential bias	Manuscript, Discussion
Interpretation	20	Give a cautious overall interpretation of results considering objectives, limitations, multiplicity of analyses, results from similar studies, and other relevant evidence	Manuscript, Discussion
Generalisability	21	Discuss the generalisability (external validity) of the study results	Manuscript, Discussion
**Other information**			

Funding	22	Give the source of funding and the role of the funders for the present study and, if applicable, for the original study on which the present article is based	Funding

*Give information separately for cases and controls in case-control studies and, if applicable, for exposed and unexposed groups in cohort and cross-sectional studies.

Note: An Explanation and Elaboration article discusses each checklist item and gives methodological background and published examples of transparent reporting. The STROBE checklist is best used in conjunction with this article (freely available on the Web sites of PLoS Medicine at http://www.plosmedicine.org/, Annals of Internal Medicine at http://www.annals.org/, and Epidemiology at http://www.epidem.com/). Information on the STROBE Initiative is available at www.strobe-statement.org.
